# Integrating Low-Cost Eye-Trackers to Enhance Design Education: A Case Study in University Course

**DOI:** 10.3390/s25165070

**Published:** 2025-08-15

**Authors:** Juan-Carlos Rojas, Juan Luis Higuera-Trujillo, Margarita Vergara

**Affiliations:** 1 School of Architecture, Art and Design, Tecnologico de Monterrey, Monterrey 64700, Mexico; jcrojasl@tec.mx; 2 Institute for the Future of Education, Tecnologico de Monterrey, Monterrey 64700, Mexico; 3 Department of Mechanical Engineering and Industrial Design, University of Cadiz, 11519 Puerto Real, Spain; 4 Department of Mechanical Engineering and Construction, Universitat Jaume I, 12071 Castelló, Spain; vergara@uji.es

**Keywords:** eye-tracker, higher education, educational innovation, low-cost, design process

## Abstract

The integration of technology in the classroom should be based on low-cost devices and affordable solutions, allowing educators to fully explore their potential benefits. Product design education is undergoing a profound transformation in response to these changes. The aim of this study is to demonstrate the integration of low-cost eye-tracking (ET) technology within a product design process. This research presents a practical case involving a group of design students who incorporated an ET device, as well as an alternative tracking method (AT) that simulates eye movement, to develop a product following a custom design methodology. The impact of both the methodology and the low-cost technology was evaluated through surveys administered to forty-seven students. The evaluation focused primarily on “utility, novelty, and relevance” as key aspects. The results showed consistently high approval ratings for both technologies. However, ET received significantly higher and more favorable evaluations. A detailed analysis of the evaluated elements indicated a strong preference for ET in terms of utility, novelty, and relevance. Furthermore, a correlational analysis revealed that students associated the integration of low-cost technology with usefulness and a positive experience. The findings of this case study highlight that low-cost devices and innovative methodologies are effective tools for enhancing teaching and learning experiences for students, educators, and researchers.

## 1. Introduction

The education field is currently undergoing a profound transformation, poised at the brink of a paradigm shift. Educators should cultivate an environment that encourages reciprocal exchange, dependence, and action, but major changes involve significant investments. Improving skills, capabilities, and other elements in students requires multiple factors that include primarily technology [1]. The ongoing changes in the academic landscape are exerting a direct impact on various disciplines, with design being at the forefront of this transformative process. Over the past few years, the advantages of integrating design education (D.E.) into different areas of study have become increasingly evident. When viewed as a formal and professional activity, design activity emerges as a key differentiator that resonates across a wide array of professional fields, including architecture, fashion, graphics, product design, and engineering. In these domains, the cultivation of design skills finds meaningful connections with science and technology, contributing to the enhancement of innovative and creative practices [2].

In contemporary discourse, the concept of design is increasingly perceived as a multifaceted process undertaken by an agent to devise solutions based on various factors, including external environments, objectives, desired structures, and properties stipulated by numerous requirements and specifications [3,4,5]. Consequently, the D.E. is undergoing a transformative shift, adapting to the dynamic demands of the present educational landscape. Certain limitations have been detected, particularly regarding technological inclusiveness. It is worth nothing that according to Rivera-Chang [6], the current design pedagogy still partially relies on century-old technology, processes, and materials. Moreover, educational institutions have not yet integrated complex processes, resources, and investment demanded to adequately support high-level design projects [7]. In recent times, there has been a discernible shift in the approach taken to tackle this issue. Several researchers have referred to a novel perspective on practice and pedagogy, which emphasizes research and technological applications [8]. Consequently, teachers, universities, and related faculties have embarked on promoting an interconnection between three fundamental elements in D.E.: an authentic pressure context, an innovative pedagogy, and novel technologies application. However, to advance a renewed perspective on education, scientific research, and project-based problem solving [9,10,11], recourse investment is needed, while simultaneously creating new and strong connections between industry and stakeholders [12]. In light of this ongoing transformation, it is imperative to advance concrete strategies that drive the evolution of D.E. Key areas of opportunity include the redesign of curricula aligned with emerging competencies, the purposeful integration of technologies, the adoption of scalable low-cost solutions, and the promotion of pedagogical approaches that foster greater student motivation and meaningful engagement.

### 1.1. New Curriculum in Design Education

One of the primary challenges inherent to this study lies in substantiating whether the integration of the employed low-cost technology effectively underpins the requisites for altering student learning paradigms. Presently, it is sought through curriculum transformation, aiming to provide all students with the requisite knowledge, competencies, and attributes imperative for success, thus catalyzing novel challenges [13]. A curriculum that systematically contemplates enhancement unfolds avenues for students to embark upon a trajectory toward professional prowess; however, this is subject to high costs. There exists a necessity to integrate novel practices [14]; although there is still no consensus on which specific practices should be prioritized in product design, it is acknowledged that design students tend to develop distinct identities, engage actively, and work intuitively, with a strong inclination toward industry involvement—an aspect that demands greater resources. To support these goals, a curriculum incorporating co-development, transdisciplinary collaboration, and emerging technologies [15] is recommended. Some examples of such curricular changes already exist. To illustrate recent developments, we can consider those undertaken by universities in North America and Latin America. Sanders [16] asserts that design schools persisting in teaching traditional practices fail to mirror the industry’s evolution, emphasizing that there is a need that has to be met at high costs. Naveiro and de Souza Pereira [17] note that graduates are increasingly required to possess technical knowledge, analytical aptitude, and technological mastery. In either case, a curriculum that seeks to incorporate courses that integrate problem-solving thematic content [18], a pioneering pedagogy [19], and strategies that promote deeper and more adaptive learning using technology [20] will be one that advances toward a brand-new design educational design landscape [20,21,22].

### 1.2. Eye-Tracking Technology

This research aims to explore the potential of a tool closely linked to the design discipline, with the intention of contributing to emerging shifts in educational approaches and strengthening key disciplinary elements. Presently, eye-tracking (ET) technology integration in design discipline, development, and execution of projects has emerged as a prominent trend [23,24,25,26]. ET technology application contributes significantly to amplifying the design dynamics and facilitating the developmental process. The intrinsic worth of this technology resides in its capacity to discern gaze behaviors. Rooted in the scrutiny of ocular movements, ET technology furnishes an objective metric of the locus of an individual’s attention, both in conscious and subconscious contexts [27,28]; established in the principle of eye movement, holds significant relevance in comprehending users’ visual attention patterns. The benefits of this tool have led to its increasing adaptation in various facets, including education [29]. Speculatively, ET technology integration into the designer’s curriculum has the potential to catalyze a form of learning intricately linked to the facets that technology empowers us to comprehend. It is not surprising that technology can reshape educational aspects, consistently showcasing elements like playability, reliability, utility, richness, quality, and relevance [30,31] of the applied technology in recent years. Furthermore, it transforms courses by considering the scalability, sustainability, and effective practices of the applied technologies [32]. Notably, the technology selected for this study has features that can be useful, enriching, novel, and relevant to students; an example of this is the precise semiotic encapsulation of symbols [33], the spatial arrangement of objects [34], the capacity of the gaze to interact with the environment and its multifaceted processes [35], and adjacent processes that are more complex such as attention, decision-making, and facets of memory [36]. For design professionals, the utility of this technology can manifest in heightened awareness of the intricacies of visual attention, shedding light on elements capable of eliciting interest and influencing user decision-making processes [37,38,39].

### 1.3. Low-Cost Eye-Trackers

The integration of technology into education faces significant challenges, particularly in balancing its technical capabilities, pedagogical benefits, and future costs. One of the key considerations in advancing this integration is determining whether low-cost devices present a viable alternative. A decade ago, Ferhat and Vilariño [40] provided an overview of low-cost eye-trackers, highlighting the advantages of conventional technology and offering a promising perspective for multi-activity applications. However, even at that time, it was acknowledged that achieving an optimal cost–benefit balance remained crucial for successful implementation [41]. Over the past decade, concerns have persisted regarding the accuracy and speed of low-cost solutions, ranging from modified webcams and open-source tools [42] to more specialized algorithms [43]. Despite these limitations, this enthusiasm has driven further exploration of cost-effective alternatives, fostering the development of affordable hardware for research across various fields [44]. In particular, we have focused on a low-cost device that has been examined in multiple relevant studies. Gazepoint^®^ trackers have demonstrated acceptable accuracy and precision in various experiments; however, their systems remain relatively basic [45]. Additionally, their effectiveness in more complex investigations requires further improvements, particularly in terms of pressure sensitivity [46]. Despite these limitations, low-cost devices such as Gazepoint^®^ offer key advantages, including portability and adaptability beyond laboratory settings [47]. With appropriate methodological precautions, these factors will be carefully considered in this research. Other examples that illustrate the impact of our research include brands that have begun to integrate visualization capabilities with eye-tracking technologies, which are already present in both educational settings and industry [48,49]. In both contexts, the use of low-cost devices consistently raises questions about the efficiency of their application and calibration for data capture. In recent years, low-cost systems have continued to gain attention as research tools, with notable improvements in hardware accuracy [50]. Although this progress is closely tied to the refinement of methodologies and the careful selection of participants for testing [51], recent studies have addressed relevant topics [51,52], serving as valuable references for our research, which aims to explore the use of these trackers in creative [53] and educational contexts when applied within a defined methodological framework.

### 1.4. Enhancing, Motivation and Eye-Tracking

Finally, another challenge of this research is to contribute to the integration of low-cost technology at the forefront of D.E. courses and curricula. It is essential to recognize that the adoption of pioneering technologies and innovative teaching methods can lead to profound transformations—ranging from generational adaptation and emotional factors [54] to new processes of understanding, assessment, retrospection, and reflection on the part of students [55]. These changes aim to foster motivation and confidence in students’ adoption and acceptance of new approaches. The use of low-cost technologies such as eye-tracking (ET) and similar tools is well documented [56], and students often engage in assessments to obtain valid metrics and explore their integration with other cognitive measures [57,58]. In recent years, fostering emotional engagement and creativity among students has become a growing focus of attention. For this reason, the implementation of project-based methodologies that incorporate design approaches and low-cost technologies has shown promising results in the development of new university curricula [59]. Although, as previously mentioned, high-end ET equipment offers greater technical advantages, recent advances suggest that low-cost technologies based on similar techniques have made it feasible to replicate such dynamics [56]. Thus, we find ourselves at a point where the development of strategies that effectively integrate these technologies, design processes, creative skills, and emotional components [60] is essential for fostering more engaging and dynamic educational experiences.

## 2. Materials and Methods

### 2.1. Objective

The main objective of this research is to contribute to the understanding of the integration of low-cost technologies in product design processes within higher education. Throughout the development of a design project within a curricular course, students will apply a design methodology specifically created to demonstrate the use of low-cost technology to achieve innovative outcomes. For this purpose, the following research questions (RQs) have been formulated to guide the key focus areas that support this contribution to the pedagogical paradigm through the use of such technologies:

RQ1: How did the students perceive the pedagogical methodology during the course experience?
RQ2: How did students assess the experience of low-cost technology?
RQ3: How did the students assess the utility, novelty, and relevance components of the low-cost technology?

### 2.2. Participants

A total of 47 students participated in this study. Thirty-two were women and 15 were men, with an average age of 22.57 years (SD = 1.016). The students were in their final year of the four-year Industrial Design program. The sample was obtained from the course “Design Products and Systems Two”, taught on three occasions: Fall–Winter 2020, Spring–Summer 2021, and Fall–Winter 2022. Two of these courses used the same technology (ET_n_ = 21 women, 8 men) and the other (AT_n_ = 11 women, 7 men). There were no criteria for selecting the groups of students on each course. All participants were provided with an informed consent form explaining the objectives, procedures, potential benefits, and guarantee of data confidentiality from the monitoring of their projects. The courses were taught at Tecnológico de Monterrey, Monterrey Campus, Mexico.

### 2.3. Methodology

The study presented in this article is a field study that comprises the following components: (1) participants (design students); (2) a project and its product design methodology utilizing low-cost ET technology, (3) data collection tools to assess aspects of the developed product.

#### 2.3.1. Design Project Methodology

**Figure 1:**
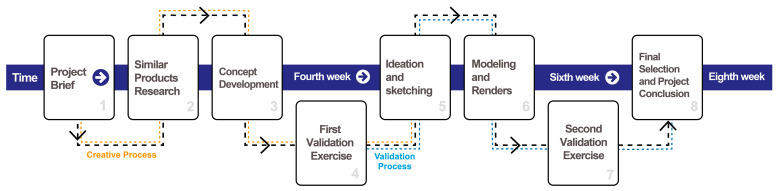
The DPMT scheme.

A project-based methodology was developed as a key component of the curricular innovation implemented in the aforementioned courses. This methodology, referred to as the Design Project Methodology with Technology (DPMT), was structured to incorporate a low-cost technology as a tool for validating the products designed by students. The DPMT was developed following the phases of design thinking [60,61,62,63], which served as its reference framework. Students engaged in product design experience accompanied by the design of simple research protocols through two experiments embedded in specific phases. Over eight weeks, with approximately five to six hours of class per week, students developed their projects. One of the participant groups completed the final two weeks of the DPMT in an online format. As a result, they were unable to use the low-cost ET device; however, an alternative tracking (AT) method, supported by different technological means, was employed. DPMT phases and the overall structure is presented in Figure 1.

**Figure 2:**
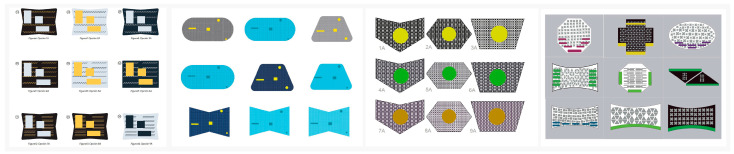
Example of concept development.

**Figure 3:**
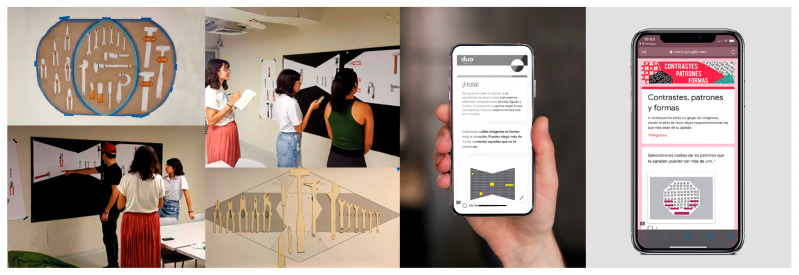
Example of initial validation exercises, low-resolution mock-ups (**left**), example of online surveys (**right**).

**Figure 4:**
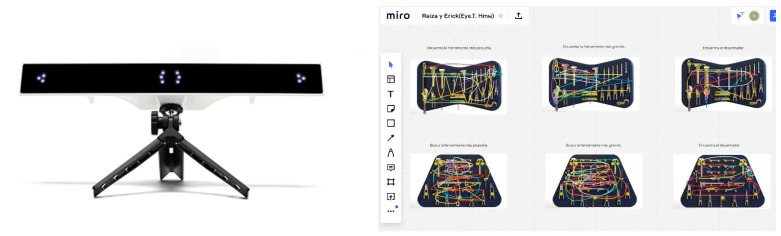
GP3 HD Eye-tracking Bar by Gazepoint^®^ (**left**). MIRO^©^ Online board (**right**).

1. Project brief: The design challenge was introduced during the course sessions, along with constraints related to format, time allocation, and execution phases. The project brief tasked students with designing an innovative tool board intended to enhance the organization and accessibility of tools within predefined visual boundaries and maximum dimensional limits.
2. Research into similar products: A preliminary search was carried out to identify existing products and various models of tool boards. Following this, a detailed analysis was conducted to evaluate the strengths and weaknesses of the identified products.
3. Concept development: A creative process was designed with a focus on visual perception, drawing on principles from Gestalt theory and related applications [64,65,66]. Students created a series of tool board archetypes using basic shapes, geometric silhouettes, and contrasting colors. In total, approximately 25 to 27 boards were developed (see Figure 2).

4. First validation exercise (validation without ET/AT technology): The validation process was conducted using physical tool board prototypes and an online survey (see Figure 3). Here, students were tasked with designing a validation protocol, which involved elements such as survey design, scheduling participant sessions, timed tool search tasks, and the use of threads to trace participants’ search trajectories as they located items on the prototype boards. This phase took place during the fourth week of the project.

5. Ideation and sketching: The student teams selected three proposals based on the most relevant data from the previous phase. Subsequently, new design sketches were developed to include more detailed features of the tool boards, facilitating internal feedback sessions with the teachers.
6. Modeling and renders: Following the previous phase, each student team selected the tool board proposal they deemed most appropriate for advancing within the methodology. The chosen designs were then modeled and rendered using professional software, including Autodesk Fusion 360^®^ (https://www.autodesk.com/products/fusion-360/, access on 23 June 2025) and KeyShot^®^ (https://www.keyshot.com/). The renderings were carefully produced to ensure photorealistic quality, accurate proportions, and a neutral background; essential criteria for the JPG images required in the subsequent phase of the DPMT.
7. Second validation exercise (validation with ET/AT technology): The next phase took place during the sixth week and involved the use of technology. Students repeated the process of creating their research protocol, but in this case, it was designed for the use of the tools and their selected boards. As previously noted, two student groups participated: one group used ET technology in the classroom, while the other employed an AT method in an online setting. The ET device used was the GP3 HD (Version 5.3.0) by Gazepoint^®^, Vancouver, BC, Canada (https://www.gazept.com/), a low-cost eye-tracking system operating at 150 Hz, based on Gazepoint’s proprietary platform for eye movement quantification (see Figure 4, left). The AT method utilized the MIRO^©^ platform, Amsterdam, Netherlands (www.miro.com), which incorporated mouse tracking [58] to simulate eye movements during the online activity (see Figure 4, right).

**Figure 5:**
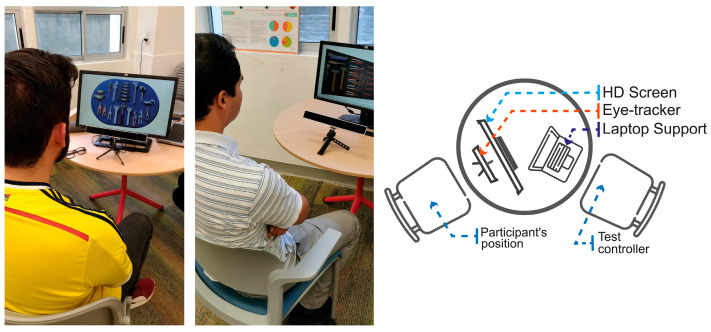
Setup of the ET application space.

The second validation experience of the DPMT was carried out by the student teams using both technologies. The same dynamics from the initial non-technological validation were replicated. As part of the evaluation protocol, participants were asked to search for tools, with the tracking of search time and movement trajectories. However, in this phase, the data were collected directly through the ET or AT platforms. Students invited faculty members and fellow students to participate in the evaluation protocol. For the ET application, Figure 5 shows the setup for the use of ET equipment. This configuration follows a traditional arrangement, with the screen and sensor bar positioned at the bottom, maintaining a distance of approximately 50–70 cm from the participant. In the case of the online group, students used Zoom^®^ to conduct the evaluation using the AT method.

**Figure 6:**
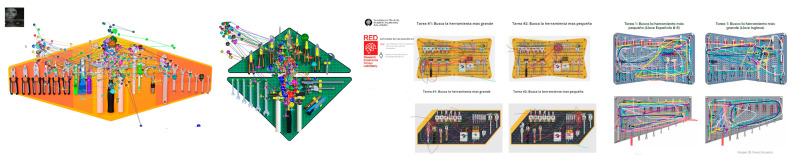
Example of material for analysis and decision-making.

In both cases, data were collected from at least 35 participants, consisting of qualitative measures (time in seconds and search trajectories) for locating items on the selected board. Using this data, students conducted basic statistical analyses, reviewing the results for searches involving large tools, small tools, or any items of particular interest. They also visually examined the images generated by the low-cost tool platforms (see Figure 6). Subsequently, the student teams shared their data with peers to analyze and compare results. This stage enabled students to make informed decisions on modifications and improvements to the boards in preparation for presenting their final proposal.

**Figure 7:**
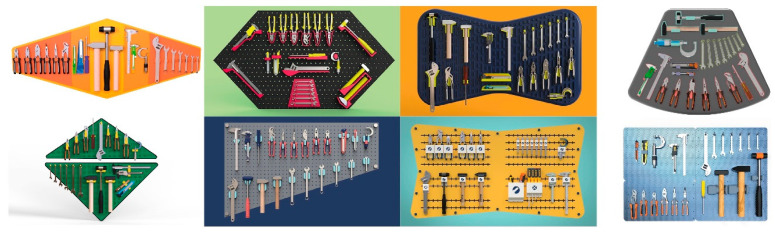
Examples of tool boards designed by students’ teams using DPMT.

8. Final selection and project conclusion: The student teams concluded the project with end-of-course activities, all of which took place during the final week of the course. The project culminated in a comprehensive report that detailed the entire DPMT process and highlighted the most effective tool board design proposed by each team. Figure 7 presents several of the tool boards developed by students’ teams.

#### 2.3.2. Assessment Instrument

Upon completion of the project, a survey was conducted to gather students’ experiences with using the DPMT to design their tool boards. The survey was designed to explore two aspects of the experience described in this study. The first focused on students’ experiential perceptions, while the second aimed to assess key components that inform the development of the proposed pedagogical methodology. Accordingly, the survey was divided into two sections. The first section consisted of six questions: which low-cost technology was used during the DPMT; the student’s gender; three questions regarding students’ overall experience and background; and one question addressing their perception of the different phases experienced throughout the project. Below, the final four questions of this section are presented, along with their multiple-choice format and corresponding response options:

1. Do you consider that you have practiced a complex or robust product design process during your degree? (a) Yes, (b) No, (c) I am not sure.
2. Do you consider that the use of technology or evaluation enhances your product design process? (a) Yes, (b) No, (c) I am not sure.
3. Do you consider the traditional design process is enough or does adding ET technology improve the design process? (a) Traditional, (b) Both, (c) I am not sure.
4. Do you indicate at which stages of this design process the technology used may have an impact? (a) Exploration/Ideation, (b) Definition/Design (c) Develop/Tests, (d) Deliver/Feedback.

The second section consisted of three questions on three key components of interest to our research. The first component is the “utility” of the DPMT in the experience of the process. This component asked about the need to know if the technology incorporated in the pedagogical innovation was perceived as it was intended, something previously studied in the incorporation of technology [30,31,67]. The second component is “novelty”. This component asked about the need to know if the embedded technology presented any novelty for the students in their design process. Since the novelty of technology is always an important aspect of motivation and acceptance [31,68]. The third component is “relevance”. This component was asked to complement the students’ perception of the relevance of the technology incorporated in their design process. This component, like the previous ones, is closely linked to the student’s acceptance and good experience [31,69,70]. The following three questions addressed the components of utility, novelty, and relevance, using a whole-number Likert scale ranging from 1 to 5. Participants rated each item on this 5-point scale, where 1 indicated *strongly disagree* and 5 indicated *strongly agree*, with higher values reflecting a stronger level of agreement and a more favorable evaluation of each component.

5. How do you evaluate the utility of technology in the experienced design process?
6. How do you evaluate the novelty of technology in the experienced design process?
7. How do you evaluate the relevance of technology in the experienced design process?

## 3. Results

All the statistical analyses were performed with the SPSS statistical software SPSS (IBM SPSS Statistics for Windows, Version 27.0, IBM Corp: Armonk, NY, USA). The three analyses are described below.

### 3.1. General Experience

**Figure 8:**
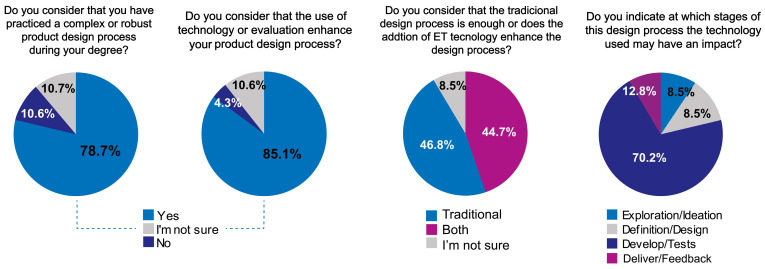
Percentages of answers from the students’ survey.

The initial analysis of the student survey responses offers valuable insights into the first four questions. Results from the first item showed that 78.7% of students believed they had previously engaged in a complex design process. The second question revealed that 85.1% of participants considered the DPMT to have reinforced their knowledge of the design process. The third question explored the students’ acceptance of the DPMT, yielding mixed responses and indicating varying levels of adoption. Notably, the fourth question focused on the perceived clarity of DPMT’s role in validation processes. In this case, 70.2% of students regarded the methodology as a useful framework for both development and testing activities. These findings are visually represented in Figure 8.

### 3.2. The Impact of Three Key Components

The results of the second survey section unveiled an assessment of the three key components. The results indicated utility (*M* = 4.72), novelty (*M* = 4.57), and relevance (*M* = 4.68). All three components received favorable evaluations. The descriptive statistics are displayed in Table 1.

### 3.3. The Effect of Experience with Technology

To evaluate students’ perceptions of technology use during the DPMT process, the data were initially checked for normality and consistency. An independent samples *t*-test (two-tailed) was then conducted to compare the responses of students who used ET and those who used AT. Due to the small and unequal group sizes, statistical power was verified using G*Power 3.1.9.7 [71,72], with the significance level set at *p* = 0.05. The results revealed significant differences across the three evaluated components: utility, novelty, and relevance. Participants in the ET group reported significantly higher evaluations of utility (*M* = 4.97, *SD* = 0.19) compared to the AT group (*M* = 4.33, *SD* = 0.77), *t*(45) = 4.27, *F* = 63.44, *p* < 0.001. Regarding novelty, the ET group also obtained higher scores (*M* = 4.83, *SD* = 0.47) than the AT group (*M* = 4.17, *SD* = 0.79), *t*(45) = 3.62, *F* = 9.45, *p* = 0.004. For relevance, the ET group again showed higher ratings (*M* = 4.90, *SD* = 0.31) than the AT group (*M* = 4.33, *SD* = 0.84), *t*(45) = 3.29, *F* = 40.39, *p* < 0.001. The effect sizes calculated using Cohen’s *d* were large in all cases: 0.95 (utility), 0.93 (novelty), and 0.90 (relevance), supporting the impact of technology on students’ experience. A complete table of data is presented in Table 2.

Finally, a correlation analysis was conducted to explore the relationship between the use of ET or AT technology and students’ evaluations of the key components of the DPMT design process, as well as their perception of traditional versus technology-enhanced design. The question “Do you consider that the traditional design process is sufficient, or that adding electronic technology improves the design process?” was treated as an ordinal variable, coded as follows: (a) Traditional = 1, (b) Both = 2, (c) Not sure = 3. This coding captured a spectrum of positions reflecting different levels of openness to technology use in the design process. Spearman’s correlation analysis revealed significant relationships between the type of technology experience and students’ evaluations of utility (*r* = −0.56, *p* < 0.001), novelty (*r* = −0.49, *p* < 0.001), and relevance (*r* = −0.41, *p* = 0.004), confirming the trends observed in previous analyses. Additionally, a significant correlation was found between technology experience and students’ perceptions of the sufficiency of traditional design (*r* = −0.34, *p* = 0.019), suggesting that students who used ET were more likely to value the integration of electronic technology in the design process compared to those who used alternative tools. These findings provide further evidence of the impact of technology on students’ perceived experience during the implementation of the DPMT. A complete table of data is presented in Table 3.

## 4. Discussion

The objective of this case study was to demonstrate and improve the integration of low-cost ET technology into a design course. By observing experience in a curricular course, students applied a novel methodology alongside low-cost technology to achieve innovative outcomes. The findings provide valuable insights into the effectiveness of integrating emerging technologies into design education and their potential to enhance students’ learning experiences. We will approach this discussion by attempting to describe our findings and answering the questions in our research questions. Firstly, the student experience, our RQ1. The deployment of our methodology entails many elements that must be considered, especially for educators who want to apply our methodology. Our RQ1 on the perception of the pedagogical methodology is exposed in this case study. Students’ perceptions of the pedagogical methodology used in this study were generally positive. A majority of 78.7% recognized the complexity and robustness of the design process they followed, while 85.1% agreed that the integration of technology enhanced their experience. Building our methodology on a structure that is familiar to the students, we were successful in adopting it. These results indicate that DPMT was well received and perceived as a valuable enhancement to traditional D.E. Our findings align with several ideas that we want to highlight of RQ1 posed. The role of active, technology-supported learning methodologies in D.E. is evident [8,63]. DPMT was structured around design thinking principles [62,63], incorporating phases of research, ideation, validation, and refinement. This structured approach is consistent with studies highlighting the importance of iterative, hands-on learning experiences in design disciplines [16,61]. By allowing students to test and validate their designs using objective, data-driven feedback mechanisms, DPMT aligns with best practices in evidence-based design education [12,15]. Moreover, the positive reception of the methodology reinforces prior findings that technology-enhanced design education, in our case low-cost, fosters deeper student engagement [5,9]. Specifically, ET technology provides an empirical framework for evaluating visual attention and usability, enabling students to adopt a more analytical, research-oriented approach to product validation [27,35]. However, students divided opinions on the sufficiency of traditional design methods suggest that additional pedagogical refinement may be necessary. While technology integration enhances student learning outcomes [13,57], research also indicates that not all students readily adapt to complex methodologies [31,70].

Following up on RQ2, regarding students’ experience with low-cost technology, one of the most significant findings of this study was the positive evaluation of the students’ experience with low-cost technology, regardless of which of the two technologies presented (ET or AT) they used. A comparative statistical analysis showed that students rated the ET more favorably than the AT across all assessed aspects (see Table 2). However, this was not intended to exclude or negatively highlight either technology; on the contrary, both were well accepted. Nevertheless, our primary interest lies in understanding how ET was perceived. These results reveal ET as a more useful, engaging, and effective tool compared to the AT. These findings align with prior research on educational technology acceptance, which suggests that students respond more positively to technologies that provide real-time and interactive experiences [31,32]. Something important to mention here, the preference for ET technology over AT can be attributed to the novelty and disruption of ET technology. In contrast, AT technology, which relied on mouse-based tracking, was perceived as less effective for the project. This aligns with previous findings that cursor-based methods do not fully replicate gaze behavior, leading to lower precision in attention mapping [51,58]. Additionally, AT participants worked in an online setting, where engagement levels may have been lower due to limited interaction [57]. Research on learning technologies in D.E. suggests that students are more engaged when technology enhances their analytical processes [10,30]. Students’ strong preference for ET is consistent with studies that indicate how dynamics similar to the use of ET enhance their ability to learn and improve their critical processes in product design [34,39]. Also, students exposed to eye-tracking technology develop stronger analytical skills, particularly in user-centered design approaches [25,54]. 

Finally, the RQ3 is an assessment of utility, novelty, and relevance elements. The fundamental objective of this study was to examine how students perceived the utility, novelty, and relevance of low-cost ET technology in the design process. The results from the survey revealed strong positive evaluations across all three dimensions (see Table 1 and Table 2). These findings are consistent with research on technology acceptance and engagement in education, where students tend to favor tools that provide tangible benefits (utility), introduce new learning paradigms (novelty), and align with real-world applications (relevance) [30,31]. The high utility rating suggests that students perceived the integration of ET technology as a valuable enhancement to their design process. This aligns with studies emphasizing the importance of novel methodologies in design education, where objective performance metrics improve decision-making and validation processes [27,39]. By incorporating these evidence-based evaluation techniques, students were able to refine their product designs based on real user interactions rather than relying solely on subjective feedback. Furthermore, the correlation analysis revealed that students who rated technology as highly useful also tended to find it novel and relevant, reinforcing the interdependence of these three key dimensions in low-cost technology adoption [32,67]. The novelty rating indicates that students viewed eye-tracking as an innovative addition to their design workflow, distinguishing it from traditional validation techniques. Previous studies have shown that introducing novel technologies into the curriculum fosters deeper cognitive engagement, particularly when students perceive the tools as cutting-edge and professionally relevant [29,69]. The positive novelty ratings in this study suggest that ET was perceived as a breakthrough tool for design validation, offering students a new perspective on user behavior analysis [23]. The relevance rating indicates that students saw a clear connection between ET technology and its applicability in professional design practice. This aligns with the tendency of professional activities, where ET has been widely adopted for usability testing, cognitive psychology, and more [27,28]. These findings are consistent with constructivist learning theories, which emphasize the importance of real-world relevance in education [57]. When students perceive a direct link between their projects and professional practice, they are more likely to engage deeply and retain knowledge for future applications [18,48].

### Limitations and Future Work

This case study has provided several findings on the incorporation of low-cost ET technology and our methodology. However, this study has some limitations that should be considered when interpreting the results and designing future research. The first consideration is the sample size and generalizability. The study was conducted with a sample of 47 students from a single Industrial Design course at Tecnológico de Monterrey. While this provides valuable insights into students’ experiences with low-cost ET technology, the results may not be generalized to other institutions, disciplines, or broader educational contexts. Future research should expand the sample size to include multiple universities, diverse design fields (e.g., graphic design, architecture, UX design), and different educational levels. The second consideration is the comparison between ET and AT Technologies. The study showed ET and AT experiences, but differences in engagement may have influenced students’ perceptions. ET was used in a controlled classroom environment, providing a structured experience with technology. AT was used in a moment for online format, where students had limited physical interaction. Future research should standardize validation conditions in DPMT to ensure a more balanced experience in both technologies. The third consideration is technological accessibility and cost. This case study focused on showing this implementation, primarily, with low-cost ET technology, there are still accessibility concerns with this technology. Some universities may lack the financial resources to integrate hardware-based ET systems into their curricula. Future work should explore more scalable and open-source alternatives, such as webcam, other ET devices, or explore alternatives with Artificial Intelligence. The final consideration is the pedagogical adaptation and training needs. Our findings showed that most students perceive low-cost technology positively, but some were still unsure of its relevance. This suggests that more refinement is needed from a pedagogical perspective for the following: “briefing sessions or exercises before using the technology” or “Contrasting mixed methodologies that further combine design and ET dynamic” or “Create didactic material for future projects with ET technology”. Future studies should continue refining these dimensions to foster broader, more inclusive, and pedagogically sound applications of ET in creative learning environments.

## 5. Conclusions

Our study highlights the potential of low-cost technology, mainly ET, as an accessible and effective tool for enhancing design education. By integrating DPMT into a university course, students were able to participate in a structured, technology-enhanced design process that combined traditional ideation techniques with novel validation methods. A key finding of this study is that students responded positively to the low-cost ET technology, particularly in terms of utility, novelty, and relevance. The significantly higher ratings for ET over AT suggest that real-time gaze tracking provides more valuable insights for product validation than cursor-based alternatives. These results align with contemporary trends in evidence-based D.E., where objective analytical tools enhance students’ ability to assess user interaction and optimize their design product decisions. The low-cost nature of technologies is a fundamental aspect of their applicability; this study contributes to showing how affordable solutions can effectively support D.E. when connected with a well-structured pedagogical basis. Looking forward, a key challenge lies in conducting longitudinal studies to assess the long-term effects of low-cost eye-tracking on the development of competencies and emerging skills. Future work will focus on identifying scalable, affordable alternatives and fostering interdisciplinary collaboration to support the paradigm shifts demanded by D.E. Incorporating accessible and innovative technologies into design curricula holds significant potential to strengthen students’ critical thinking, creativity, and technological literacy in an ever-evolving educational context.

## Figures and Tables

**Table 1:** Descriptive statistics for key components.

Question	Mean	Std. Dev.
How do you evaluate the utility of technology in the experienced design process?	4.72	0.579
How do you evaluate the novelty of technology in the experienced design process?	4.57	0.683
How do you evaluate the relevance of technology in the experienced design process?	4.68	0.629

Note: Ratings based on a 5-point Likert scale, where 1 = Strongly Disagree and 5 = Strongly Agree.

**Table 2:** Descriptive and statistical and T-test results for ET and AT experience.

Key Components Questions	Factor	N	Mean	Std.Dev.	t	F	Sig.
How do you evaluate the utility of technology in the experienced design process?	ET	29	4.97	0.186	4.268	63.439	**<0.001**
	AT	18	4.33	0.767			
How do you evaluate the novelty of technology in the experienced design process?	ET	29	4.83	0.468	3.622	9.447	**0.004**
	AT	18	4.17	0.786			
How do you evaluate the relevance of technology in the experienced design process?	ET	29	4.90	0.310	3.285	40.386	**<** **0.001**
	AT	18	4.33	0.840			

Note: Ratings based on a 5-point Likert scale, where 1 = Strongly Disagree and 5 = Strongly Agree. Statistical significance was set at *p* = 0.05.

**Table 3:** Correlations between elements in the student experience.

	How Do You Evaluate the Utility…	How Do You Evaluate the Novelty…	How Do You Evaluate the Relevance of Technology…	Do You Consider That the Use of Technology … Product Design Process …	ET or AT Experience
Do you consider that the use of technology … product design process …	**0.019**	0.201	0.211	-	0.270
ET or AT experience	**0.000**	**0.000**	**0.004**	0.270	-

## Data Availability

The data used to support the findings of this study can be made available by the corresponding author upon request.
